# Mitochondrial DNA Phylogeography of the Norway Rat

**DOI:** 10.1371/journal.pone.0088425

**Published:** 2014-02-28

**Authors:** Ying Song, Zhenjiang Lan, Michael H. Kohn

**Affiliations:** 1 Department of Ecology and Evolutionary Biology, Rice University, Houston, Texas, United States of America,; 2 The State Key Laboratory for Biology of Plant Diseases and Insect Pests, Institute of Plant Protection, Chinese Academy of Agricultural Sciences, Beijing, China; 3 Key Laboratory of Weed and Rodent Biology and Management, Institute of Plant Protection, Chinese Academy of Agricultural Sciences, Beijing, China; BiK-F Biodiversity and Climate Research Center, Germany

## Abstract

Central Eastern Asia, foremost the area bordering northern China and Mongolia, has been thought to be the geographic region where Norway rats (*Rattus norvegicus*) have originated. However recent fossil analyses pointed to their origin in southern China. Moreover, whereas analyses of fossils dated the species' origin as ∼1.2–1.6 million years ago (Mya), molecular analyses yielded ∼0.5–2.9 Mya. Here, to study the geographic origin of the Norway rat and its spread across the globe we analyzed new and all published mitochondrial DNA *cytochrome-b* (*cyt-b*; N = 156) and D-loop (N = 212) sequences representing wild rats from four continents and select inbred strains. Our results are consistent with an origin of the Norway rat in southern China ∼1.3 Mya, subsequent prehistoric differentiation and spread in China and Asia from an initially weakly structured ancestral population, followed by further spread and differentiation across the globe during historic times. The recent spreading occurred mostly from derived European populations rather than from archaic Asian populations. We trace laboratory strains to wild lineages from Europe and North America and these represent a subset of the diversity of the rat; leaving Asian lineages largely untapped as a resource for biomedical models. By studying rats from Europe we made the observation that mtDNA diversity cannot be interpreted without consideration of pest control and, possibly, the evolution of rodenticide resistance. However, demographic models explored by forward-time simulations cannot fully explain the low mtDNA diversity of European rats and lack of haplotype sharing with their source from Asia. Comprehensive nuclear marker analyses of a larger sample of Norway rats representing the world are needed to better resolve the evolutionary history of wild rats and of laboratory rats, as well as to better understand the evolution of anticoagulant resistance.

## Introduction

Mitochondrial DNA (mtDNA) analyses of some widespread Murine rodent pest species, such as the Roof rat (*Rattus rattus*), pacific rat (*R. exulans*), and the house mouse (*Mus musculus* ssp.), have enabled inferences of the geographic origins and population structure of their archaic populations, and have pointed towards some likely pre-historic and historic routes by which these invasive species have colonized parts of the globe [1]–[6]. Moreover, in the case of the house mouse, the study of the phylogeographic history of the species has been an important step towards an improved understanding of the ancestry of laboratory mouse strains [7], [8]. This is because an understanding of population structure and sub-specific status is important to more effectively use the laboratory mouse as a biomedical model, and to generate new strains of the laboratory mouse through the collaborative cross and similar such efforts that aim to capture more of the previously untapped wild genetic variation of the mouse [7], [8]. Both the rats and house mice adopted a commensal lifestyle as they took advantage of the aggregation of humans in settlements and developing agriculture. Thus, by reconstructing their spread informative connections of (pre-) historical human migrations and major geopolitical events with the geographic distributions of rodent mtDNA haplotypes have been discovered [1]–[6].

The Norway rat (*R. norvegicus*) is as about as geographically widespread as is the Roof rat and the house mouse; however, while the other species have been studied with molecular markers to some detail [1]–[6], the evolutionary history and global spread of the Norway rat have not been studied so far. Similarly, the Norway rat is the wild ancestor of the laboratory rat. However, as has been the case until recently for the laboratory mouse; the ancestry of laboratory strains of the rat is poorly understood [9]. With regard to the latter statement it is known that genetic diversity is reduced, but it is not known what the contributions of various wild populations to the laboratory strains are. Finally, what to our knowledge has not been tested in any of the globally distributed rodent pest species is whether rodent eradication attempts by humans have affected mtDNA diversity?

Eastern Central Asia, including today's northern China and Mongolia, long has been considered as the area where the Norway rat has originated [10]–[12]. However, recent analyses of dated fossils, judged to represent ancestral forms of the Norway rat, indicated that the species originated in today's Southwestern China, ∼1.2–1.6 million years ago (Mya) [13], [14]. The analysis of rat fossils collected in the Choukoutien Cave in northern China further indicated that the species arrived there ∼0.14 Mya and was widely distributed in most of China and adjacent Asian countries ∼0.01–0.13 Mya [14].

During the Medieval Ages (15^th^ century) Norway rats began their spread across the globe [15]. Anecdotal reports of the species' presence in Europe date to the mid 16^th^ century; evidence considered as more reliable dates to the early 18^th^ century [15]. The Norway rat apparently reached North America by the middle of the 18^th^ century [15] and Africa by 19^th^ century [16]. The final wave of spread of the species across the globe likely began with the intensification of human naval activity, which we would expect to be an ongoing process that affects the global phylogeographic structuring of the Norway rat.

Conceivably, molecular data will be informative with regard to an improved understanding of the origin, diversity, and spread of wild Norway rats. Such analyses could improve the understanding of the origin of the laboratory rat and of its genetic diversity relative to its wild ancestors. Molecular data may provide first important clues about the potential effect rodent pest eradication efforts had on mtDNA diversity. Unfortunately, little genetic information on the Norway rat is available for analysis, and the data published is scattered across various sources. Moreover, mtDNA data on European Norway rats are virtually absent. Here we provide an analysis of the mtDNA sequence data comprised of all published *cytochrome-b* (*cyt-b*) and D-loop sequences of wild Norway rats and select inbred strains, as well as newly determined sequences of Norway rats from Europe (Germany and France) [17], [18].

In addition to a general analysis of mtDNA phylogeography of the Norway rat we analyze the mtDNA data with respect to the evolution of resistance to anticoagulant rodenticides such as warfarin [18], [19]. Such resistance has become known shortly after the introduction of rodenticides in the 1950s, and has been reported in Europe, North America and other continents [20]–[22]. The vitamin K 2,3-epoxide reductase subunit 1 (*Vkorc1*) has been identified as the major resistance gene [23], [24]. Various single nucleotide polymorphisms (SNPs) in *Vkorc1* cause resistance [25], [26]; the most prominent such mutation alters the amino-acid Y at position 139 to C (Y139C), which is highly abundant and well-studied in countries such as Germany [25], [26]. In this study we analyzed the mtDNA variation of rats from Europe carrying the Y139C mutation with regard to diversity and distribution of particular mtDNA haplotypes with two aims. First, we examined whether mtDNA haplotypes that are presently common in Europe were already present in other populations; foremost the presumed archaic populations from Asia. Second, we compare mtDNA diversity in European populations with the overall diversity of the Norway rat to obtain a measure for the change in genetic diversity that may be due to chemical pest control and the evolution of resistance.

Here we report that the mtDNA-based study of wild Norway rats can reveal details about the evolution and spread of the species. Select patterns of haplotype sharing observed between geographic areas seem to be explained by recent human geopolitical events. Our study hints at the difficulties to trace the precise geographic origins of laboratory rats but documented their origin from mostly derived European and North American populations that harbor a fraction of the natural diversity of the species. Moreover, we show that the neutral processes of drift and founder events only insufficiently explain the dramatic lack of haplotype sharing between ancestral and derived warfarin resistant Norway rat populations. Our study points to the need to sample the Norway rat more comprehensively across the globe and to analyze the species for nuclear marker diversity.

## Methods

### Sequence Data compilation

**Figure 1:**
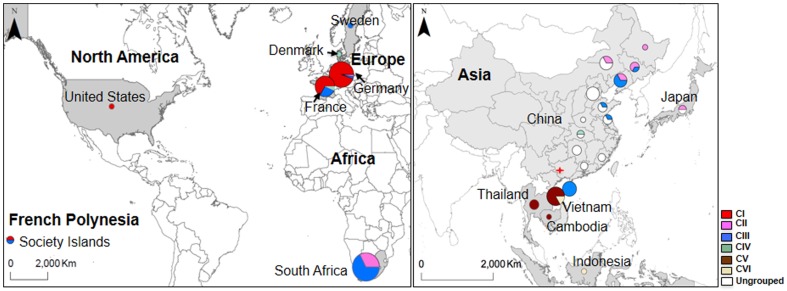
Continents and countries (shaded in grey) sampled for wild Norway rats (circle size reflects sample size *N*; c.f.Tables 1 & 2) and observed frequencies of *cyt-b* haplotype groups (clades CI-CVI). The red star indicates the location where the earliest Norway rat fossils were found ∼1.2–1.6 Mya [13], [14]. The sampling sites (provinces) in China from north to south are Heilongjiang, Jilin, Liaoning, Inner Mongolian, Hebei, Shandong, Henan, Jiangsu, Hubei, Hunan, Fujian, Guangdong, Yunnan and Hainan. The figure was drawn using ArcMap using ArcGIS 10.1 [53]

This study relied in large part on a collection of published mtDNA sequences of the Norway rat. Specifically, we study 664 base pairs (bp) of the mtDNA D-loop and 549 bp of the mtDNA *cyt-b* gene, representing, respectively, 212 and 156 wild Norway rats from 9 and 12 countries representing Asia, Europe, Africa, and North America, as well as French Polynesian Islands (Fig. 1; Tables 1 & 2, S1 for sample details; Genbank accession Nos. JX887160-JX887174). From NCBI (http://www.ncbi.nlm.nih.gov/) we downloaded 40 D-loop (278–664 bp) and 105 *cyt-b* sequences (549 bp) of wild Norway rats, as well as 27 D-loop (664 bp) and five *cyt-b* (549 bp) sequences of inbred strains. For phylogenetic analyses we included *cyt-b* sequences of 11 other *Rattus* spp., 2 *Niviventer* spp., 2 *Berylmys* spp., 3 *Bandicota* spp. and 4 *Mus musculus* subspecies, and D-loop sequences of 4 other *Rattus* species (Table S1). To obtain matching sequences for geographic regions not previously studied with mtDNA we newly sequenced the D-loop for 172 Norway rat samples from Germany and France collected during pest control conducted in the mid 1990s [17], [18]. For 51 samples representing different D-loop haplotypes we sequenced *cyt-b*.

**Table 1:** Geographic occurrences and frequencies of *cyt-b* haplotypes C1–39.

	N =	C1	C2	C3	C4	C5	C6	C7	C8	C9	C10	C11	C12	C13	C14	C15	C16	C17	C18	C19	C20	C21	C22	C23	C24	C25	C26	C27	C28	C29	C30	C31	C32	C33	C34	C35	C36	C37	C38	C39
Europe	53	43	1	6	1	1	1																																	
Denmark	1						1																																	
France	15	9		4	1	1																																		
Germany	36	34	1	1																																				
Sweden	1			1																																				
Asia	63		1	5				1	6	1	2	6	1	1	1	1	1	4	1	4	1	1	1	2	1	2	1	1	1	1	1	8	1	1	2	1	1			
Cambodia	1																																		1					
China	47			5					6	1	2	6	1	1	1	1	1	4	1	4	1	1	1	2	1	2	1	1	1	1	1									
Indonesia	1																																				1			
Japan	2		1					1																																
Thailand	3																															2				1				
Vietnam	9																															6	1	1	1					
Africa	37		12	22																																		2	1	
South Africa	37		12	22																																		2	1	
North America	1	1																																						
USA	1	1																																						
French Polynesia	2	1		1																																				
Society Islands	2	1		1																																				
Inbred strains	5		1	1																																				3
All	161	45	15	35	1	1	1	1	6	1	2	6	1	1	1	1	1	4	1	4	1	1	1	2	1	2	1	1	1	1	1	8	1	1	2	1	1	2	1	3

Sample size (N = )

**Table 2:** Geographic occurrences and frequencies of D-loop haplotypes D1–25.

	N =	D1	D2	D3	D4	D5	D6	D7	D8	D9	D10	D11	D12	D13	D14	D15	D16	D17	D18	D19	D20	D21	D22	D23	D24	D25
Europe	174	123	25	1	3	6	8	3	1	1	1	1	1													
Denmark	1												1													
France	17	11				4					1	1														
Germany	155	112	25	1	3	1	8	3	1	1																
Sweden	1					1																				
Asia	32					1								1	5	1	7	4	8	1	1	1	2			
China	30														5	1	7	4	8	1	1	1	2			
Japan	1					1																				
Vietnam	1													1												
Africa	1					1																				
Egypt	1					1																				
North America	2	1		1																						
USA	2	1		1																						
French Polynesia	3	2				1																				
Society Islands	2	1				1																				
Tubai	1	1																								
Inbred strains	27			12		1																		6	6	2
All	239	126	25	14	3	10	8	3	1	1	1	1	1	1	5	1	7	4	8	1	1	1	2	6	6	2

Sample size (N = )

### Molecular methods

Genomic DNA of newly sequenced Norway rats was prepared from liver tissue using the DNeasy tissue kit (Qiagen, Valencia, CA, USA). The *cyt-b* of rats from Germany/France was amplified using published primers [27]. D-loop sequences of rats from Germany were amplified using the forward and reverse primers (5′- TCA GGA CAA TCA AGA AGA AGG A -3′ and 5′- TGA GGG TAG GCA AGT AAA GAG G-3′; designed using Primer 3 [28]). M13 sequencing tails (5′- CACGACGTTGTAAAACGAC-3′ and 5′- GGATAACAATTTCACACAGG-3′) were attached to the 5′-ends of forward and reverse primers. D-loop sequences of rats from France were amplified using published primers [27].

PCR thermo-cycling conditions generally were as follows: 2 minutes at 94°C, followed by 30 cycles of 30 seconds at 94°C, 30 seconds at 56°C, 1 minute at 72°C, and a final 5 minutes at 72°C. Products were cleaned using ExoSAP-IT (USB, Cleveland, OH, USA), sequenced in both directions (Sanger method), and read on an ABI Prism™ 3730xl DNA (Applied Biosystems, Foster City, CA, USA). Reads were assembled, proofread and aligned to the corresponding regions of the mtDNA of *R. norvegicus* (GenBank No. NC_001665) using the software Lasergene 7.2 [29]. Missing bases of shorter sequences were treated following the minimal distance rule [30].

### Data analysis

#### Haplotype Tree and Network Reconstruction

First, the phylogeny of *Rattus* and the relationships between *cyt-b*/D-loop haplotypes of the Norway rat were reconstructed using Bayesian Markov Chain Monte Carlo (MCMC) runs over 1,000,000 generations as implemented in MrBayes 3.1 [31], and a 50% majority rule tree was constructed after burn-in of an initial 2,000 trees. The nucleotide substitution model (HKY+I+G) was chosen by the algorithm implemented in the Modeltest 3.7 software [32] for both D-loop and *cyt-b* sequences.

Second, haplotype relationships were reconstructed in a different fashion from above to estimate the time since the origin and differentiation of clades (in millions years ago; Mya) using the software package BEAST (v1.7.0.) [33]. We assumed 10.4–14.0 Mya as the divergence time between *Mus* and *Rattus* [1]. As above, Modeltest chose the HKY+I+G substitution model and an uncorrelated lognormal relaxed-clock model for both the D-loop and *cyt-b* data; the latter were partitioned into 3 codon positions. Bayesian MCMC analyses were run for 30,000,000 steps with posterior sampling every 1,000 steps after burn-in of an initial 3,000 trees. The software package Tracer v1.5 [34] was used to evaluate the stability of all parameters and to visualize the trace files generated in BEAST.

Third, we depict haplotype relationships as Median-Joining (MJ) networks generated by using the software Network 4.6 [30].

Finally, we used two approaches to reconstruct the likely past geographic distributions (expressed as probabilities) of the presently observed mtDNA haplotypes. We first conducted Bayesian Binary MCMC (BBM) analysis for 10 million runs with a burn-in of 10,000 steps using default settings in the software package RASP v2.0b RASP [35]. Then we used a likelihood approach implemented in the software package LAGRANGE v. 20120508 that models a dispersal-extinction-cladogenesis process (DEC) [36]. The input tree for both reconstruction methods was adopted from the Bayesian analysis using BEAST software as described above.

#### Genetic Diversity and Demographics

When permitted by sample size, the population parameters nucleotide diversity (π), haplotype diversity (h. d.) and haplotype number (h) were estimated in DnaSP v5.0 [37]. Analysis of Molecular Variance (AMOVA) was done as implemented in Arlequin v3.5 [38].

The frequencies of observed pairwise nucleotide differences between mtDNA haplotypes were plotted against the corresponding expected frequencies to yield mismatch distributions using DnaSP v5.0 [37]. We obtained the expected distributions by coalescent simulations for stable and expanding populations using diversity measures as estimated from the observed sequence data in DnaSP v5.0 [37]. Goodness-of-fits between observed and expected distributions were tested using 1,000 bootstrap replicates as implemented in Arlequin v3.5 [38]. For further demographic model testing we estimated the following composite population parameters: the sum of squared deviations (SSD) [39], Harpending's raggedness index (RI) [40], Ramos-Onsins & Rozas's R2 [41], Fu's Fs [42] and Tajima's D [43]. Each of these estimators and their significance levels (obtained by 1,000 coalescent simulations) were calculated as implemented in Arlequin v3.5 [38] and DnaSP v5.0 [37].

**Figure 2:**
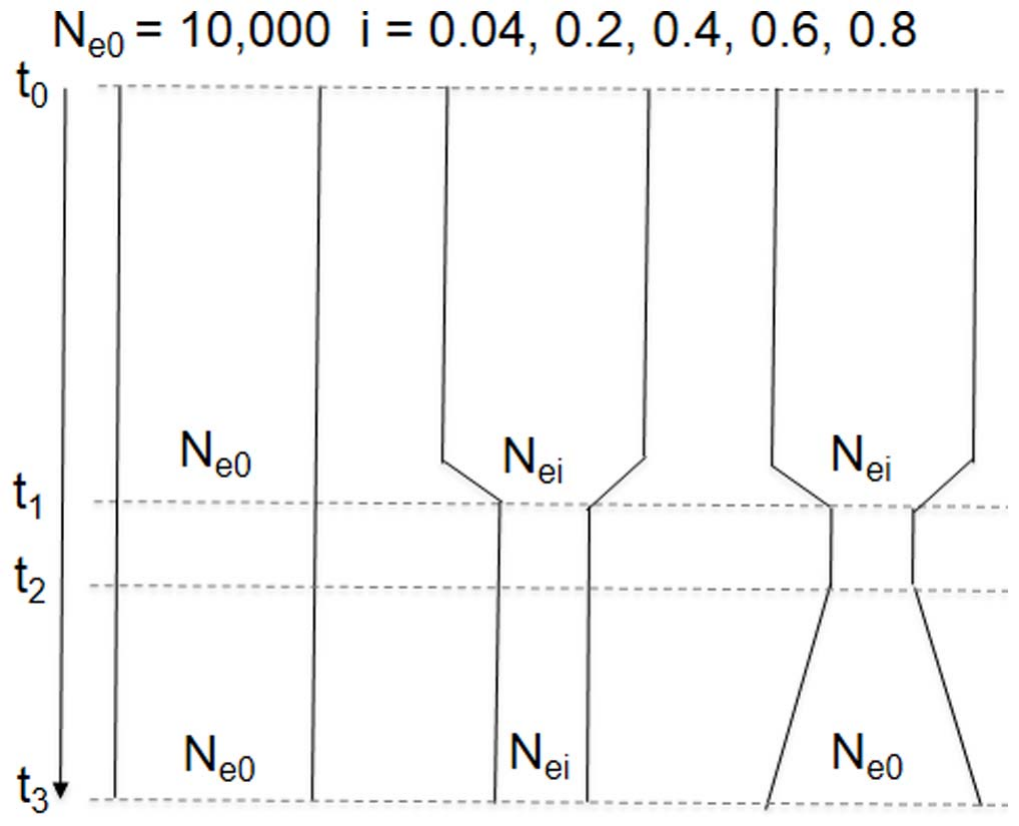
Depiction of forward simulation models and key parameters considered. We model D-loop haplotype frequencies by random genetic drift in a neutrally evolving population with an effective population size (N_e0_) of 10,000 for 250 years, assuming that the Norway rat colonized Europe at t_0_∼1750 A.D. [16] and assuming 2 generations per year [54] (left). At t_1_∼1950, or 400 generations later, we model the introduction of warfarin in form of a genetic bottleneck because initially rodent control induced high rates of mortality [55] (middle and right). For the reduction to N_ei_ we introduced 4 levels of N_ei_ after rodent control (100, 500, 3,000, 7,000) to reflect the effectiveness of warfarin in controlling rats when warfarin was introduced in the 1950s, and we assumed that rodent control remained effective by keeping N_e_ reduced (middle). Finally, we evaluate a model that assumes that resistance has evolved within 10 years (t_1_∼t_2_) [20] and constant population size growth recovers to N_e0_ (right); the evolution of resistance was modeled on one autosomal locus (representing Y139C), which freely recombines with mtDNA and is under balancing selection with coefficient of 0.3 [55]. Simulations were run for 1,000 replicates using a series of initial haplotype frequencies (*i*) for one most common haplotype (*i* = 0.04, 0.2, 0.4, 0.6 and 0.8) at the time rats settled in Germany, with the remaining haplotype frequencies equally shared by all the other haplotypes (here: 24). We assumed that a population colonized Europe that carried all 25 D-loop haplotypes detected in this study. Finally, we calculated the probability that the expected frequencies of all the 25 haplotypes are equal or greater than the observed frequency, which refers to the haplotype frequency of 0.72 for the most common haplotype (D1; see main text) in Germany.

We conducted forward-time population genetic simulations in the SimuPoP environment [44] to explore potential demographic scenarios (Fig. 2) that could explain observed mtDNA haplotype frequencies in our sample of rats from Germany, Europe, many of which are warfarin resistant.

## Results and Discussion

### Origin and spread of the Norway rat

#### Reciprocal monophyly of the Norway rat

Phylogenetic analyses that consider mitochondrial DNA haplotype polymorphisms of the Norway rat potentially improve our understanding of the relationship amongst *Rattus* [1], [45] and could reveal previously unrecognized deep coalescent events within the nominal species *R. norvegicus* that merit taxonomic review. Similar such analyses have revealed deep coalescent events within the Roof rat that prompted taxonomic revisions [1]. However, it is not known if mtDNA polymorphism data for the Norway rat would justify similar such taxonomic revisions.

**Figure 3:**
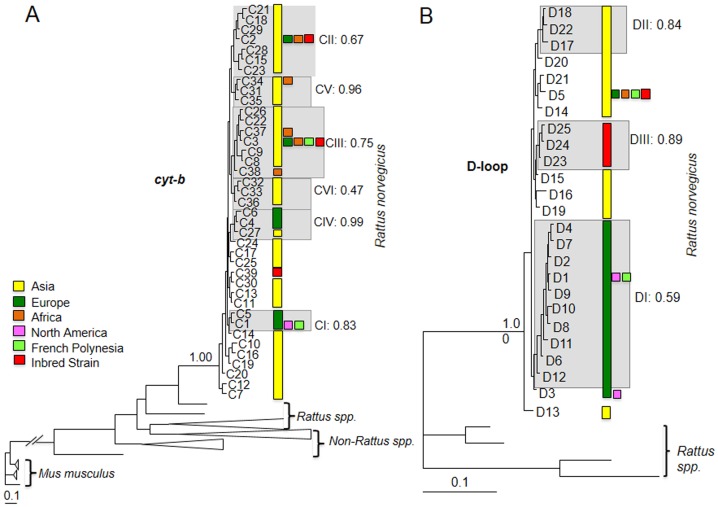
Bayesian reconstruction of *cyt-b* (A) and of D-loop (B) haplotype trees. Colored rectangles mark the sampling location. Gray shaded areas labeled with roman letters (CI-CVI) mark clades discussed in the main text. Bayesian posterior possibilities (cut-off >0.5) are shown after the roman letters. Scale bar shows number of substitutions per nucleotide.

Bayesian analyses of *cyt-b* (posterior possibility = 0.99) and D-loop sequences (posterior possibility = 1.00) confirmed the reciprocal mtDNA monophyly of the Norway rat (Fig. 3) [1], [12]. We consider none of the divisions in the haplotype tree as major (see below), and thus, unlike it was the case for the Roof rat and the house mouse, taxonomic refinements in terms of species or subspecies designations appear to be unnecessary in the case of the Norway rat.

Species miss-classification can be a problem during genetic analyses of rodent tail clips shipped between the field and molecular laboratories [46]. The observed monophyly of the Norway rat supports that 1) the newly collected sequences and other (published) sequences used in this study stem from Norway rats, and 2) that future studies on Norway rats could use mtDNA-based analyses to confirm the species under study.

**Figure 4:**
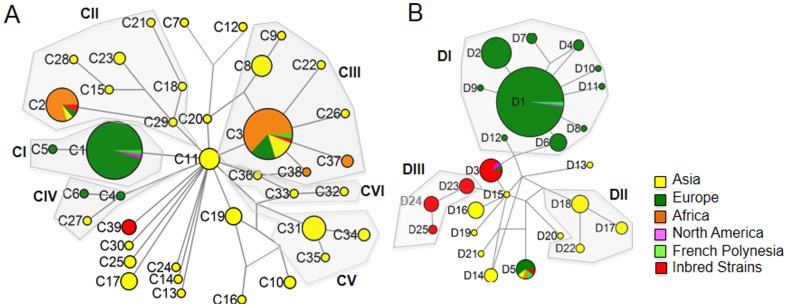
Median-Joining (MJ) mtDNA *cyt-b* (A) and D-loop (B) haplotype networks. Circle sizes are proportional to the frequency of each haplotype in the total sample. The sections of pie chart represent the relative sample size of rats from different continents. Gray shaded areas (CI-CVI; DI-DIII) mark clades as referred to in Fig. 3.

Molecular clock-based analysis of *cyt-b* sequences indicated that the Norway rat emerged ∼1.3 Mya (Fig. 4). This estimated timing of the Norway rat's origin matches up well with the ∼1.2–1.6 Mya reported for the earliest fossils of the species found in today's southern China [14]. Sequence saturation in *cyt-b* likely renders our estimate an underestimate. Despite the good fit between our molecular time estimation and the analysis of fossils, the large confidence interval of our estimate (0.44–2.35 Mya) reflects the uncertainty associated with such estimates, partly due to species tree/gene tree issues. In fact, our estimates of the timing of the origin of the Norway rat overlap with the range of previous estimates of speciation times [1]; [45]; [47]. Our work and previous work based on mtDNA sequences appear to be limited in their resolution, and thus, analyses of nuclear DNA is needed to better resolve the timing of the origin of the Norway rat as a species.

#### Intra-specific lineage diversification of the Norway rat pre-dated global population expansion

The Norway rat is about as widely distributed across the globe, as is the congeneric Roof rat. The Roof rat has spread across the globe from deeply structured archaic populations that pre-historically formed in Asia [1]. Comparison between these superficially similar commensal species might reveal commonalities and differences.

Inspection of structure and support of the intra-specific *cyt-b* haplotype tree revealed weak Bayesian support for any major clades with deep coalescent times (posterior possibilities <0.50; Fig. 3A), but supported five shallow clades (CI-CV; posterior possibilities 0.63–1.00; Fig. 3A). Clades CI-V were distinguished in the *cyt-b* MJ network (Fig. 4A). Similarly, phylogenetic analysis of D-loop haplotypes revealed poorly resolved clades DI-DIII (posterior possibilities <0.50; Fig. 3B). These clades were distinguished in the MJ network also (Fig. 4B). The poorly resolved *cyt-b* and D-loop mtDNA haplotype trees are consistent with weak intraspecific differentiation by mutation.

Molecular clock analyses based on the *cyt-b* data (D-loop data were too limited to be used) yielded divergence times of clades I-V as ∼0.10–0.20 Mya (middle/late Pleistocene). These estimates should be viewed with great caution, as there is only weak statistical support for any of these haplotype groups. However, these dates suggest that the origin of mtDNA intraspecific diversity of the Norway rat by mutation pre-dated the colonization of the globe by the species. Consequently, as not enough time has elapsed for sufficient new mutations to occur we expected to attribute any global maternal population structure discussed below to be mainly due to the sampling and loss of mtDNA lineages due to drift during dispersal; foremost founder events.

In the following we refer to the poorly supported mtDNA haplotype clades by their names mostly to facilitate discussion.

#### “Out of China” population expansion within Asia

The analysis of fossils has provided evidence for the origin of the Norway rat in the eastern parts of Central Asia, with some controversy surrounding the question whether the species originated in the northern parts of today's China and Mongolia rather than the southern regions of China.

Inspection of the *cyt-b* haplotype tree revealed that the rat samples from Asia, especially from today's China, represent the most archaic population. The *cyt-b* haplotypes from this region occupy the external and most of the internal branches of the haplotype tree (Fig. 3A). Similarly, despite much more limited sampling of D-loop sequences, haplotypes from outside of Asia are nested within haplotype groups found in Asia; particularly those found in China (Fig. 3B).

Inspection of the *cyt-b* haplotype network structure and haplotype sharing between populations revealed that haplotypes found in Asia, particularly in China, are dispersed throughout the network (Fig. 4A). C11, the most common haplotype isolated from China, occupies a central position in the network where it is only one mutational step away from at least 13 other haplotypes; nine of these exclusively found in China (C13–C14, C17, C19–C20, C24–C25, C29–C30). The four haplotypes C1, C3–4, C36 that are closely related to C11 represent samples collected from outside of China and/or across the globe (Fig. 4A). Due to the limited sampling of D-loop haplotypes the corresponding network is less informative with regard to the geographic origin of the Norway rat (Fig. 4B). However, haplotypes from Asia tend to form a part of the network from which a group of D-loop haplotypes emerged that is found in the heavily sampled European population but, except for D5, are not found in Asia and China (Fig. 4B).

Populations are expected to harbor their highest levels of genetic diversity in the area near or where they have persisted for the longest period of time [5]. Of the 39 *cyt-b* haplotypes identified, 32 (∼83%) are found in Asia, of which 24 (75%) are found in China (Tables 1 & 3). The distribution of D-loop haplotypes provides a less clear such signal, as we found that only 11 out of 25 haplotypes (40%) occur in Asia and 9 haplotypes (36%) in China (Tables 2 & 3). Thus, rats from Asia clearly harbor the greatest amount of diversity for both *cyt-b* and D-loop (Table 3); with most of this diversity attributable to the population from China where π is 2–4 fold higher when compared to other countries in Asia. Furthermore, the number of private *cyt-b* and D-loop haplotypes found in China and Asia is higher than in any other populations. Thus, analysis of mtDNA diversity indicated that the Norway rat has originated in Asia near today's China; a pattern already suggested by the geographic distribution of major *cyt-b* clades and haplotypes (Fig. 1).

**Table 3:** Analysis of *cyt-b* (first number) and D-loop (second number) genetic diversity measures.

Geographic origin	h	h.d.	P-h	π
Europe	6/12	0.33/0.56	3/9	1.51/1.17
Germany	3/9	0.11/0.45	0/6	0.50/0.88
France	4/4	0.60/0.55	2/2	2.36/6.42
Denmark	1/1	-/-	1/1	-/-
Sweden	1/1	-/-	0/0	-/-
Asia	32/11	0.96/0.87	28/10	6.41/1.46
China	24/9	0.95/0.85	23/9	6.37/9.03
Vietnam	4/1	0.58/-	2/0	2.23/-
Thailand	2/n.d.	0.67/n.d.	1/n.d.	1.24/n.d.
Japan	2/1	-/-	1/0	-/-
Cambodia	1/n.d.	-/n.d.	0/n.d.	-/n.d.
Indonesia	1/n.d.	-/n.d.	1/n.d.	-/n.d.
Africa	4/1	0.49/-	2/0	2.36/-
South Africa	4/-	0.49/-	2/-	2.36/-
Egypt	n.d./1	n.d./-	n.d./0	n.d./-
North America	1/2	-/-	0/2	-/-
USA	1/2	-/-	0/2	-/-
French Polynesia	2/2	-/-	0/0	-/-
Society Islands	2/2	-/-	0/0	-/-
Tubai	n.d./1	n.d./-	n.d./0	n.d./-
Inbred strains	3/5	0.70/0.72	1/3	3.28/2.87
All	39/25	0.86/0.76	34/22	4.73/7.91

Number of different haplotypes (h) and private haplotypes (P-h), haplotype diversity (h.d.), nucleotide diversity (π) x10^-3^, - not applicable (n.a.), not determined (n.d.)

**Figure 5:**
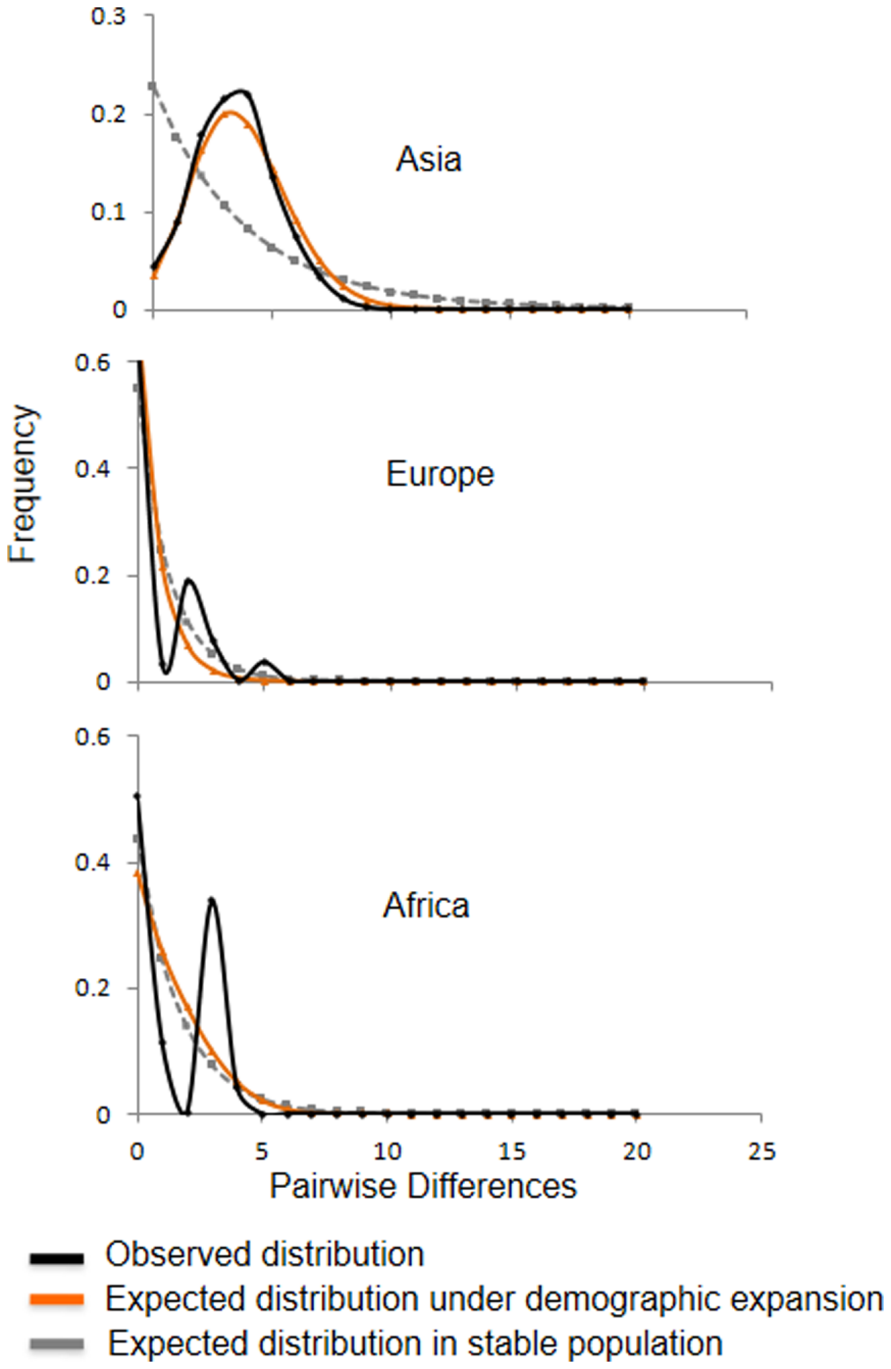
Mismatch distributions of *cyt-b* haplotypes. Expected distributions were modeled for stable populations and populations undergoing demographic expansion by using the parameters τ, θ_0_ and θ_1_ provided in Table 3.

Provided that the Norway rat has become such an abundant species, we expected to detect an expansion of its population at some time point of its demographic history, conceivably beginning in the ancestral area in Asia. The *cyt-b* mismatch distribution of haplotypes sampled from Asia deviates from a unimodal pattern (Fig. 5, top), and the other demographic statistics generally support a population expansion (Table 4). This population expansion can already be detected in the sample from China, where we observed a non-significant SSD and raggedness index, and significant values of Tajima's D (D = -1.8, p<0.05), Fs (F = -14.8, p<0.05) and R2 (R = 0.05, p<0.05).

**Table 4:** Results of neutrality tests and goodness-of-fit tests of demographic expansion for rats in selected continents based upon *cyt-b* sequences.

Continent	D	Fs	R2	SSD	RI	*τ*	θ_0_	θ_1_
Africa	0.21	1.31	0.13	0.08	0.33	2.50	0.00	1.70
Asia	−1.76*	−25.58**	0.05*	0.00	0.02	3.80	0.01	69.06
Europe	−1.61*	−1.55	0.06	0.05	0.45	3.00	0.00	0.46

D - Tajima's D.

Fs - Fu's Fs.

R2 - Ramos-Onsins & Rozas's R2.

SSD - the sum of squared deviations.

RI - Harpending's raggedness index.

Estimated demographic parameters τ, θ_0_ and θ_1_ shown as x10^−3^.

* (P<0.05) and ** (P<0.01).

Finally, to infer early stages of intraspecific differentiation due to dispersal from an ancestral population, we more closely inspected results obtained from the analysis of the samples that represent Asia and China. Specifically, we observed that none of the *cyt-b* and D-loop haplotypes sampled from other Asian countries, such as today's Vietnam, Thailand, Cambodia, Indonesia and Japan, is shared with China (Tables 1 & 2, Fig. 1). Thus, some of the earlier ‘out of China’ dispersal events followed by differentiation due to the loss of haplotypes likely occurred to Asian countries nearby China.

The comprehensive sampling of haplotypes from today's China (Fig. 1 right) enabled us to make further inferences regarding the origin of the Norway rat. Specifically, the archaic population represented by samples from China appeared to have undergone the earliest population differentiation as indicated by the number and diversity of *cyt*-b clades CII, CIII and CIV (Fig. 3A). These clades, albeit weakly supported in terms of statistical support, provide a first indication of haplotype loss due to drift and founder events (Fig. 3A). In terms of haplotype sharing Norway rats from southern China, represented by samples from the provinces currently known as Hubei, Hunan, Fujian, Guangdong, Yunnan and Hainan, differ from those in northern China represented by samples from Heilongjiang, Jilin, Liaoning, Inner Mongolia and Hebei (Fig. 1; Table S1). Thus, an early stage of population expansion and differentiation appeared to have occurred from southern China northward. This inference is in agreement with the interpretation of the fossil record of the Norway rat; as of yet the oldest Norway rat fossils were recently found in southern China.

**Figure 6:**
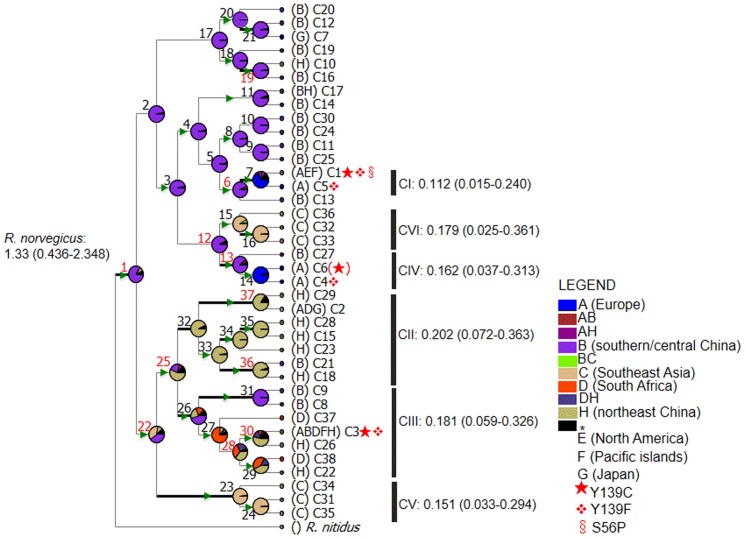
Ancestral area reconstruction based on *cyt-b* haplotypes. Pie charts on each node show the probability of each ancestral haplotype to have occurred at an inferred ancestral geographic location using the RASP method. Locations are shown in different colors and denoted with alphabetic letters A-H. Probabilities <5% were lumped together as “*”. Node numbers are shown along branches (red), indicating differences between methods (c.f. methods and Table S2). Green arrows indicate possible dispersal events. Black thick branches indicate posterior possibilities ≥0.69. Red symbols mark haplotypes found in animals carrying Y139C, Y139F, and S56P mutations in *Vkorc1*. Estimated mean divergence times and 95% highest posterior densities (in parentheses) are shown next to each of clades CI-CVI and the node supporting monophyly of the Norway rat.

Finally, we reconstructed possible past geographic distributions and frequencies of *cyt-b* haplotypes using the BBM and DEC models (see methods). The results of the two methods were congruent for 70% of the inferred nodes in terms of geographic distribution (Fig. 6, Table S2). The DEC model weakly supported a broader geographic area including China and Southeast Asia as the origin of the Norway rat (p = 0.11 at node 1; Table S2). In general, discrepancies did not affect our main conclusion that today's southern China likely is the area where the Norway rat originated (BBM, p = 0.84 at node 1), followed by at least six dispersal events resulting in clades I-IV; each with reduced haplotype diversity due to drift (Fig. 6). Reconstructed past geographic distributions of haplotypes favor a scenario where earlier dispersal events occurred in Asia, as revealed by high probabilities of assignments of ancestral haplotypes to Asia and China (Fig. 6). The development of global population structure due to sampling and random loss of lineages is apparent from the increasing probabilities of haplotype occurrences outside of Asia, and the current geographic distributions of haplotypes (see below; Fig. 1).

#### “Out of Europe” population expansion across the globe

As the Norway rat has attained a global distribution due to its affiliation with humans we expected that the process of colonization shifted from a slow natural population expansion to more rapid human-enabled dispersal. For example, we observed examples of long-range haplotype sharing between Europe and far-way locations but a general lack of haplotype sharing involving China (except C3 and D5) (Tables 1 & 2). The process of expansion and dispersal from the archaic populations in Asia to Europe remains unknown, but the near complete differentiation of the ancestral population from the derived populations in terms of haplotype sharing indicates that numerous founder events and/or massive haplotype losses during dispersal have occurred.

A key observation from inspection of haplotype trees and haplotype networks is that the globally distributed and abundant *cyt-b* haplotypes C1–C3, and the D-loop haplotypes D1–D5 belong to different clades (Figs. 3 & 4). This observation is consistent with the colonization of the globe by an initially diverse population representing much of the ancestral diversity. Moreover, it is curious that all *cyt b* and D-loop haplotypes (except C3 and D5) that are common outside of Asia are absent from the sample representing the archaic populations. These haplotypes might have been rare in the ancestral population and became frequent at a later stage as a result of founder events.

Haplotype sharing among derived populations outside of Asia (3/8 for *cyt-b* and 3/12 for D-loop) is higher than haplotype sharing between any of the derived populations and the archaic source population represented by samples from China (1/24 for *cyt-b* and 0/9 for D-loop), or even when all of Asia is considered (2/32 for *cyt-b* and 1/11 for D-loop) (Tables 1 & 2). This indicates a shift in the main source for the recent global colonization from ‘out of China’ and Asia to ‘out of Europe’.

For example, we observed interesting cases of haplotype sharing (C1 in Table 1; D1 & D3 in Table 2) between Europe and North America; continents that must have experienced much exchange since the colonization of the New World by European settlers and the importance of European mercenaries during the American War of Independence in the second half of the 18^th^ century [50].

Another interesting set of observations refers to the haplotype sharing (C1 & C3 in Table 1; D1 & D5 in Table 2) between France and the Society islands in the South Pacific Ocean; some of which have been known as French Polynesia since 1880s. Furthermore, long-range haplotype sharing (C1 and D1, Tables 1 & 2) between Europe, North America and the Society Islands may indicate recent dispersal events. Rats may have inadvertently been transported by the US Navy that used some of these islands during World War II as naval bases [51].

Norway rats might have reached the Cape Colonies, Africa, alongside Europeans. For example, during the colonial times Europeans sailed across the Horn of Africa to reach Far East Colonies for trade, and in 1652 the Dutch established the influential East India Company [15]. The Eastern Coast of Africa was part of the Silk Road maritime trading networks [48], [49]. The long-range haplotype sharing of C2, C3, and D5 between Europe and Africa (Tables 1 & 2) may be derived from Norway rats that reached Africa alongside European traders and settlers. However, haplotypes C2, C3, and D5 shared between Europe and Asia may reflect that the colonization of Africa by the Norway rat has involved Asian sources also.

In all, gene flow between continents, as measured by Fst, was 0.27 (p<0.01) between Europe and Asia, 0.38 (p<0.01) between Europe and Africa, and 0.15 (p<0.01) between Asia and Africa (data were insufficient for North America and the Society Islands). Thus, rat populations on different continents appear to be significantly differentiated from one another. However, within continents equally high or higher levels of differentiation (Fst ∼0.18–0.32) can be observed. For *cyt-b* haplotypes ∼15–38% of the variance was explained by among continent variation, whereas ∼67–85% variations were from within continent variation as revealed by AMOVA. These statistics support an important role of founder events.

**Figure 7:**
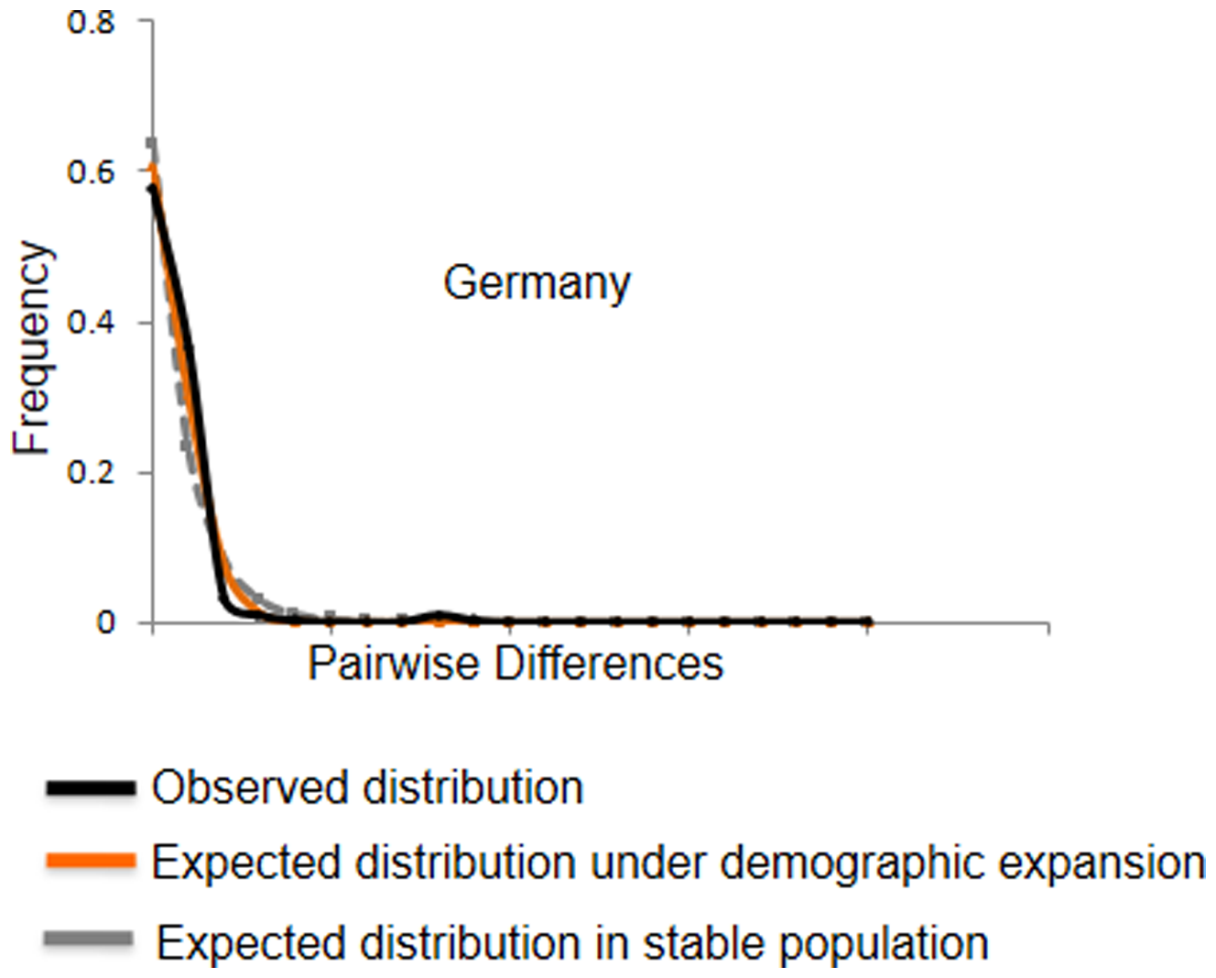
Mismatch distributions of D-loop haplotypes found in Germany. Expected distributions were modeled for stable populations and populations undergoing demographic expansion by using the parameters τ = 0.5, θ_0_ = 0 and θ_1_ = 99999.

Mismatch distribution analysis of the Norway rat in Europe reveals a multimodal mismatch distribution (Fig. 5 middle), and Fu's Fs and Ramos-Onsins & Rozas's R2 tests did not reject the null hypothesis of neutrality (Table 4). However, a significant negative Tajima's D value signifies an excess of rare alleles in Europe, which could be a result of population expansion after a founder event or bottleneck. This was further confirmed by the unimodal mismatch distribution of the D-loop region for the rats collected in Germany (Fig. 7).

In Africa the Norway rat remains sparsely distributed and often restricted to ports [16]. Mismatch distribution analysis of *cyt-b* sequences show a bimodal pattern, even though the SSD and the raggedness indices are insignificant (Fig. 5 bottom). All tests supported the null hypothesis of neutrality (Table 4). Thus, the lack of support for a population expansion indicates that the Norway rat, much unlike the Roof rat, may have difficulties establishing itself in Africa.

### Low genetic diversity due to European ancestry of the laboratory rat

The Norway rat has become an important biomedical mammalian model [49]. Varieties of the laboratory rat, such as Wistar, Sprague Dawley, Lewis rats, and others, potentially including intercrosses between inbred and wild rats were established in Europe and the US in the late 18^th^ and 19^th^ Centuries [49]. The first albino rats might have been brought from Switzerland to the United States [49].

We analyzed the mtDNA D-Loop and *cyt-b* haplotypes of 27 laboratory rat strains with respect to the wild samples. We found three *cyt-b* haplotypes and five D-Loop haplotypes in the laboratory strains (Tables 1 & 2, Figs. 3 & 4). Of these C2 and C3 are shared with wild rats that have attained global distribution, or as is the case for C39 are private (un-sampled and rare in wild rats) to laboratory rats. Similarly, the two D-loop haplotypes D3 and D5 are shared with wild rats that have attained global distribution and three haplotypes are private (D23–D25).

The pattern is consistent with the establishment of laboratory strains from derived wild lineages that already had colonized parts of the globe, notably Europe. The laboratory strains share haplotypes with wild rats collected from Europe and one haplotype (D3) with wild rats both from Europe and North America. Thus, results are consistent with proposed origins of laboratory rat strains in Europe and in the U.S.

The origin and diversity of the laboratory rat with respect to its wild cousins merits further study. In terms of haplotype number, h, the analysis of *cyt-b* and D-loop haplotypes indicated that laboratory rats represent a subset of the genetic diversity of rats that colonized Europe and North America, and an even smaller subset of the total diversity of the species (Table 3). In terms of *cyt-b* and D-loop haplotype numbers laboratory strains only capture ∼8 and 20% of the wild haplotypes, respectively. With regard to the archaic population from China laboratory rats capture only ∼13 and 56% of *cyt-b* and D-loop haplotypes, respectively. However, while haplotype numbers in laboratory strains appear to be lower than in wild rats, the fact that some *cyt-b* and D-loop haplotypes only found in laboratory strains belong to different clades in the haplotype trees (i.e. are rather divergent in sequence, Figs. 3 & 4) yielded relatively high values for the nucleotide diversity (Table 3).

In sum, genetic diversity as measured by haplotype numbers in the laboratory rat is low compared to its wild ancestor. The origin of the laboratory strains could be either in Europe or North America. Those whom established the rat as a model established the founding colonies from the lowered pool of genetic diversity of derived rat populations in Europe. In search of new biomedical models, and to increase genetic diversity that would enable finer scale mapping of trait loci, it might be advisable to include wild-derived Norway rats from diverse populations, particularly those from China. Nuclear marker surveys of wild Norway rats are needed to enable better inferences regarding the origin and diversity of the laboratory rat.

### Rodent control and mtDNA diversity

We study Norway rat populations from Europe, where anticoagulant resistance as mediated by *Vkorc1* is widespread [26], [50]. For the well-sampled populations from Germany, where Y139C predominates, we obtained 155 D-loop and 36 *cyt-b* sequences. We briefly report on Norway rats from France, where Y139F predominates [26], [51], for which we obtained 17 D-loop and 15 *cyt-b* sequences from 18 rats. A small sample of rats all sampled from one township in Germany carried S56P, a mutation not known to cause resistance (Table S1).

Human efforts to eradicate rodent pests could prominently have affected demographics, and thereby, mtDNA diversity. Europeans arguably must have continuously tried to limit rodent infestations in and around their dwellings and fields by trapping and other methods. Here we assume that only through the introduction of anticoagulant rodenticides in the 1950s were rat population sizes reduced in a notable fashion. This assumption is oversimplifying the situation as anticoagulants gradually have replaced other control methods, including acute poisons. Nevertheless, a recent population size reduction and recovery of European rats is supported by our demographic analyses of *cyt-b* data (Fig. 5 middle). Anticoagulant resistance evolved rapidly within ∼10 years at numerous locations, as was discovered first in Europe [20], and thus, resistance may explain population size recovery after population size crashes. Similarly, the mismatch distribution calculated based on D-loop sequences of rats from Germany supports a population expansion scenario, as it approximates an unimodal (Fig. 7) and, except for a significant SSD (0.01, p<0.05), yielded significant neutrality test values such as Tajima's D (D = −1.83, p<0.01) and Fu's Fs (F = −3.92, p<0.05).

**Figure 8:**
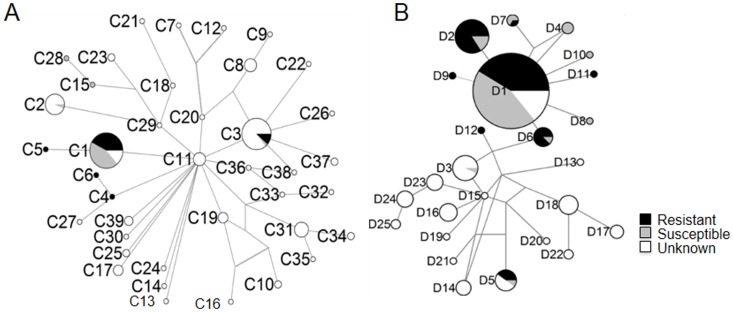
Median-Joining (MJ) mtDNA *cyt-b* (A) and D-loop (B) haplotype networks and warfarin resistance. Figure details as in Fig. 4, except that here the relative frequency of resistant and susceptible rats is shown. Only animals for which resistance status is known from experimental evidence are included here.

Diversity measures estimated for rats from Germany were extremely low but higher for rats collected from France (Table 3). We only found three *cyt-b* haplotypes (C1–C3; Table 1) in 36 rats from Germany. C1, C2, and C3 grouped with clades CI, CII and CIII, respectively, during phylogenetic- and network analyses (Figs. 3A & 8A). In France, we found four *cyt-b* haplotypes (C1, C3, C4 and C5; Table 1), which grouped with clades CI, CIII and CIV (Fig. 3A & 8A). In Germany and France C1 is the most common *cyt-b* haplotype that is shared by 94% and 60% of rats, respectively (Table 1). We found nine D-loop haplotypes (D1–D9) in 155 Norway rats in Germany (Fig. 3B & 8B). All rats carrying the common *cyt-b* haplotype C1 carry one of these seven haplotypes D1–D2, D4, D6–D9 (Table S1); the majority (∼58.8%) of these C1 haplotypes occur in conjunction with D1. For 17 D-loop sequences in France, we inferred four different D-loop haplotypes (D1, D5, D10–D11) of which ∼65% of rats share D1. It is the haplotypes C1 and D1 that have a main influence on the skew towards on abundant haplotype in the mismatch distributions obtained for rats from Europe and Germany (Figs. 5 & 7).

Only two out of five *cyt-b* haplotypes (C1, C3; Table 1) and only two out of 11 D-loop haplotypes (D1, D5; Table 2) are shared between Germany and France. The rat populations of the two countries are differentiated (Fst = 0.18, p = 0.01). An estimated 82% of this differentiation occurs at the level of local populations (AMOVA). This observation is not atypical for rodents, which tend to live in demes [52].

Warfarin resistance is found in conjunction with mtDNA haplotypes from different clades in the *cyt-b* haplotype tree (Fig. 6) and different groups of related haplotypes in the *cyt-b* and D-loop haplotype networks (Fig. 8). The vast majority of resistant rats (Y139C) carry the C1 (93%), and D1 (64%) or D2 (28%) haplotypes (Table S1). We interpret these observations as the evolution of resistance owing to mutations in the nuclear gene *Vkorc1* along one mtDNA lineage, possibly C1 and D1 or D2, and subsequent (free) recombination in a diverse population. For parsimonious reasons we prefer this scenario to a scenario that posits multiple independent origins of resistance in different mtDNA lineages. Moreover, this event likely has occurred recently as we infer from the over-representation of one *cyt-b* (C1) and one D-loop (D1) haplotype (Tables 1 & 2, Fig. 8). As neither C1 nor D1 or D2 occur in the ancestral population, i.e. are rare and presumably un-sampled, it is unclear whether these lineages carrying *Vkorc1* mutations originated in the source populations in the Far East or evolved at a later time and away from the ancestral populations. S56P, which is not known to mediate resistance, occurs in conjunction with C1 also, which suggests that nuclear genetic variation in *Vkorc1* in the C1 and D1 lineage was higher than in other lineages. The occurrences of C1 and D1 across the globe indicate that resistance seen at numerous locations of the globe has spread by human mediated long-range dispersal.

We used the observed D-loop data and forward-time population genetic simulations to examine whether under three different demographic models a mutation in an unlinked nuclear gene that causes resistance and occurred along the lineage of one initially rare mtDNA haplotype in a large diverse ancestral population (carrying 25 haplotypes, c.f. Table 2) could attain the presently observed high frequency (∼0.72 as observed for haplotype D1) in a now mtDNA genetically depleted population as currently found in Germany (Fig. 2).

Specifically, we simulated the frequency change of each haplotype under the null model of random drift in a stable neutral population (Fig. 2 left) to be compared to a model of drift in a population that is reduced in size due to rodent control (Fig. 2 middle) and a model of drift in a population that is reduced in size due to rodent control but that recovers in size due to the evolution of resistance (Fig. 2 right). In the last model we consider the observed time between the introduction of warfarin rodenticide and the first reports of resistance ∼10 years later. The latter two models likely overestimate the efficacy of rodent control with anticoagulants because these in fact only gradually entered the consumer market over the years following their introduction.

**Figure 9:**
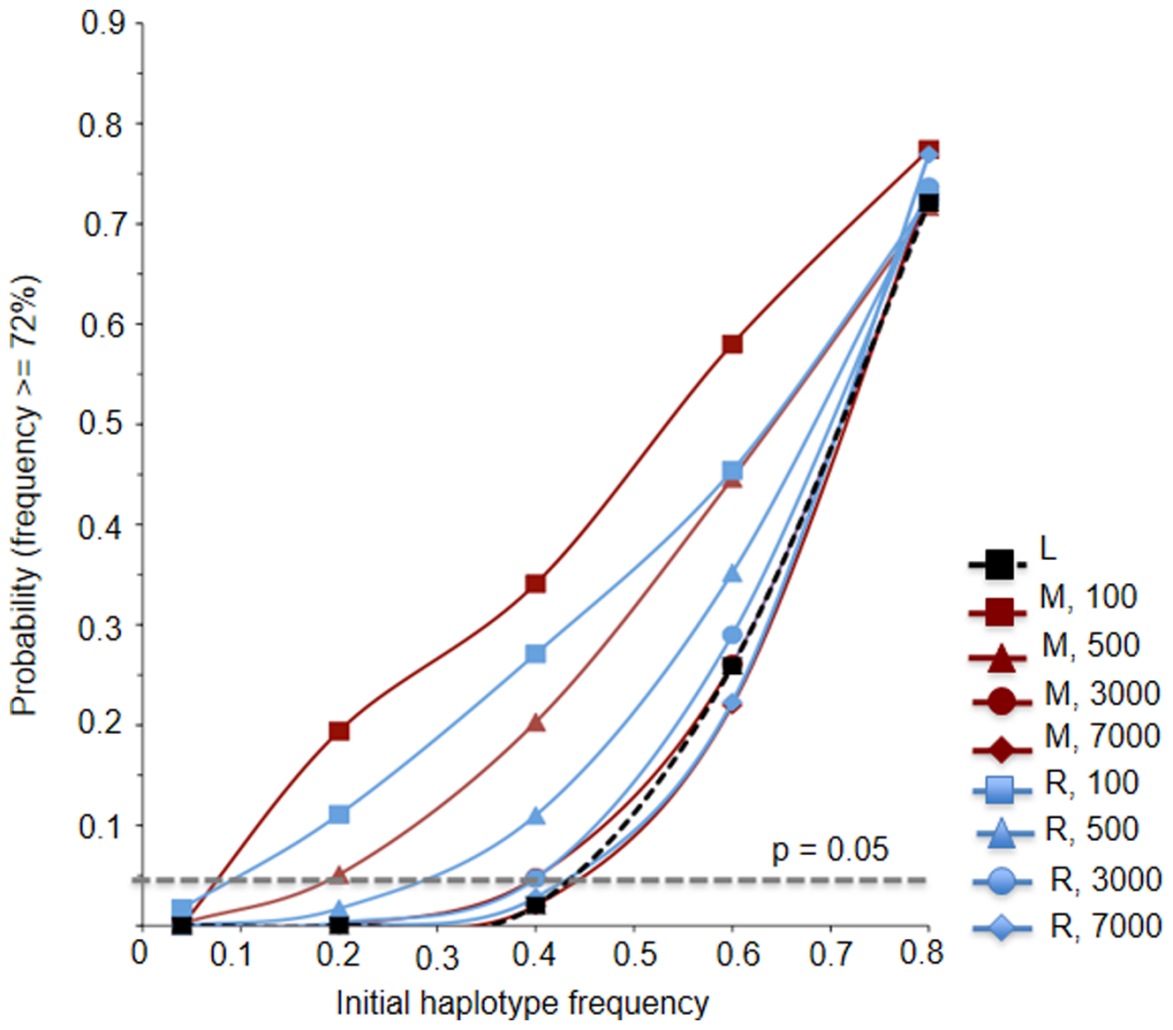
The probability that the expected haplotype frequency for the most common haplotype is equal to or greater than the observed frequency of 0.72. Shown are the models described in Fig. 2 left (L), middle (M) and right (R) considering various levels of population size reductions to N_ei_ = 100, 500, 3,000 and 7,000. The grey dashed line indicates the probability of 5% of the most common haplotype to reach a frequency ∼0.72 or higher.

The simulation results show that the probability of one haplotype to reach a high frequency is mainly determined by the initial haplotype frequency (i) and the severity of the bottleneck (N_ei_) (Fig. 9). Note the length of the bottleneck is not varied because we assume resistance has evolved about 10 years after the introduction of warfarin. We observed that an initial haplotype frequency of ∼0.45 or higher needs to be assumed for the now most common haplotype D1 to reach a frequency ∼0.72 by random genetic drift in a neutral population of constant size (Fig. 9). This scenario is unlikely because D1 (and C1) and their closely relatives such as clade I of the D-loop tree (and clade I of the *cyt-b tree*) are not found in the ancestral population from Asia, and thus, can be assumed to be rare originally. We thus reject the null model involving drift only.

We model a reduction in population size assuming various levels of mortality due to warfarin. The required starting frequency is reduced to as little as ∼0.07 to have a 5 percent chance to reach ∼0.72 if the mortality induced by warfarin is very high (99%) (Fig. 2 middle & right, Fig 9). We consider this model describing the presence of as many as 1,400×1/2N_e_ copies of the resistance allele in conjunction with an mtDNA haplotype (e.g. D1 or C1) at the time of introduction of warfarin selection as plausible at the 5% level. However, we find the model in disagreement with our working assumption that resistance should have been rare or absent from the study area. Moreover, the model is in disagreement with our assumption that the evolution of resistance should result in a recovery of population size. Acceptance of the model would require acceptance of a scenario where the presence of resistant rats in the population pre-dated the introduction of warfarin in the 1950s.

When we modeled population size recovery due to the evolution of resistance the initial frequency of the now common haplotype would need to be assumed similarly at about ∼0.1, or 2,000×1/2N_e_, when warfarin use resulted in very high levels of mortality of 99%. Thus, at the 5 percent acceptance level we consider this model as acceptable also, but consider it more realistic as a population size reduction is included.

From both of the latter models we conclude that resistance has attained a considerable frequency rapidly, and possibly predated the introduction of warfarin in our study area. At any of the higher assumed starting frequencies of the Y139C resistance allele we observed that the model describing a reduced population size (Fig. 2 right) yielded higher probabilities of observing the presently high haplotype frequency than the model describing population size reduction and subsequent recovery due to resistance (Fig. 9). It is plausible that the modeling of population size reduction and subsequent recovery is inaccurate because of the introduction of second generation anticoagulants, such as bromadiolone, difenacoum, and brodifacoum; all designed to kill warfarin resistant rats in the early 1970s, and thus, the population size recovery might have been much below of what we have modeled. Notably, runs of these models with a starting N_e_ of 100,000 did not change our conclusions, but we observed that introduction of a much earlier severe bottleneck can explain the observed data best, but did not enable us to explore the importance of population size reduction and recovery. Moreover, modeling an early population size crash essentially only leaving the currently frequent haplotypes intuitively can best explain the results, and thus, is trivial.

In sum, models involving population size reduction explain the loss of genetic diversity, and the skew towards one very common mtDNA haplotype, better than a model involving drift in a stable population. Models involving population size recovery after population size reduction performed well also. However, we failed to fully explain the observed distribution of haplotype frequencies using either model unless we assume rather high starting frequencies of the most common haplotypes, which are highly associated with the nuclear mutation Y139C that causes resistance. Our work prompts at the interesting hypothesis that resistance alleles in the German population of rats studied already had attained high frequencies prior to the introduction of warfarin rodenticide. Thus resistance could have evolved elsewhere, such as in the U.S. where warfarin was invented and used first. Given the limited amount of detail that can be inferred from the mtDNA data the study of the evolution and spread of resistance requires nuclear marker surveys.

## Supporting Information

Table S1 **The geographic origin of newly collected and published sequences.** We provide sequence length, haplotype IDs, GenBank accession numbers (and/or references) of D-loop and *cyt-b* sequences. The *Vkorc1* genotypes of samples are shown if known.
(XLS)

Table S2 **The inferred ancestral geographic areas are provided for each node shown in the** ***cyt-b*** **haplotype tree depicted in** **Fig. 6** **.** Their relative probabilities as inferred using the software packages RASP and LAGRANGE (see methods) are provided if applicable. Only probabilities >0.05 are shown. The nodes shown in underlined font indicate those nodes where we observed differences between the RASP and LAGRANGE methods.
(DOCX)
