# Supplementary material for: Mitochondrial DNA Phylogeography of the Norway Rat
Source: PLoS One. 2014 Feb 28;9(2):e88425. doi: 10.1371/journal.pone.0088425 (PMC3938417; doi:10.1371/journal.pone.0088425)
Supplement: Table S2 — The inferred ancestral geographic areas are provided for each node shown in the cyt-b haplotype tree depicted in Fig. 6 . Their relative probabilities as inferred using the software packages RASP and LAGRANGE (see methods) are provided if applicable. Only probabilities >0.05 are shown. The nodes shown in underlined font indicate those nodes where we observed differences between the RASP and LAGRANGE methods. (DOCX) [file pone.0088425.s002.docx]

**Table S2.** The inferred ancestral geographic areas are provided for each node shown in the *cyt-b* haplotype tree depicted in Fig. 6. Their relative probabilities as inferred using the software packages RASP and LAGRANGE (see methods) are provided if applicable. Only probabilities >0.05 are shown. The nodes shown in underlined font indicate those nodes where we observed differences between the RASP and LAGRANGE methods.

| Node no. | Inferred ancestral area (RASP) | Probability (RASP) | Inferred ancestral area (LAGRANG) | Probability (LAGRANG) |
| --- | --- | --- | --- | --- |
| 21 | B | 0.9748 | [B\|B] | 0.9984 |
| 20 | B | 0.9948 | [B\|B] | 0.9981 |
| 19 | B | 0.9746 | [H\|B] | 0.9051 |
|  |  |  | [B\|B] | 0.05011 |
| 18 | B | 0.9948 | [B\|B] | 0.8566 |
|  |  |  | [B\|BH] | 0.1343 |
| 17 | B | 0.9944 | [B\|B] | 0.9744 |
| 11 | B | 0.9485 | [B\|B] | 0.8531 |
|  |  |  | [B\|BH] | 0.1395 |
| 10 | B | 0.972 | [B\|B] | 0.9984 |
| 9 | B | 0.9971 | [B\|B] | 0.9984 |
| 8 | B | 0.9917 | [B\|B] | 0.9979 |
| 7 | A | 0.6813 | [A\|A] | 0.3937 |
|  | AB | 0.0959 | [AEF\|A] | 0.1815 |
|  | B | 0.0719 | [AF\|A] | 0.1727 |
|  |  |  | [AE\|A] | 0.1594 |
| 6 | B | 0.9313 | [B\|A] | 0.4841 |
|  | AB | 0.0525 | [B\|AF] | 0.08299 |
|  |  |  | [B\|F] | 0.07796 |
|  |  |  | [B\|B] | 0.07787 |
|  |  |  | [B\|AE] | 0.0771 |
|  |  |  | [B\|E] | 0.07246 |
| 5 | B | 0.9779 | [B\|B] | 0.8126 |
|  |  |  | [AB\|B] | 0.09702 |
| 4 | B | 0.9807 | [B\|B] | 0.942 |
| 16 | C | 0.9927 | [C\|C] | 0.9961 |
| 15 | C | 0.9126 | [C\|C] | 0.9753 |
|  | BC | 0.0656 |  |  |
| 14 | A | 0.9575 | [A\|A] | 0.9695 |
| 13 | B | 0.8866 | [B\|A] | 0.8679 |
|  | AB | 0.0634 |  |  |
| 12 | B | 0.9201 | [B\|C] | 0.4277 |
|  |  |  | [AB\|C] | 0.4264 |
| 3 | B | 0.9754 | [B\|B] | 0.7311 |
|  |  |  | [B\|BC] | 0.1337 |
|  |  |  | [B\|AB] | 0.05473 |
| 2 | B | 0.9868 | [B\|B] | 0.9063 |
|  |  |  | [BC\|B] | 0.04767 |
| 37 | H | 0.8414 | [ADG\|H] | 0.3949 |
|  |  |  | [DG\|H] | 0.1566 |
|  |  |  | [AD\|H] | 0.1556 |
|  |  |  | [D\|H] | 0.1158 |
|  |  |  | [G\|H] | 0.0559 |
|  |  |  | [A\|H] | 0.05368 |
| 35 | H | 0.9981 | [H\|H] | 0.9987 |
| 34 | H | 0.9965 | [H\|H] | 0.9983 |
| 36 | H | 0.952 | [H\|B] | 0.9049 |
|  |  |  | [H\|H] | 0.04976 |
| 33 | H | 0.9759 | [H\|H] | 0.8135 |
|  |  |  | [H\|BH] | 0.1738 |
| 32 | H | 0.9311 | [H\|H] | 0.3834 |
|  |  |  | [H\|DH] | 0.1217 |
|  |  |  | [H\|ADGH] | 0.09969 |
|  |  |  | [H\|ADH] | 0.06781 |
|  |  |  | [H\|DGH] | 0.06412 |
|  |  |  | [H\|AH] | 0.0606 |
|  |  |  | [H\|GH] | 0.0536 |
| 31 | B | 0.9905 | [B\|B] | 0.9948 |
| 30 | H | 0.5413 | [H\|ABDFH] | 0.4282 |
|  | DH | 0.1531 | [H\|ABDH] | 0.118 |
|  | D | 0.0699 | [H\|BDFH] | 0.1149 |
|  | AH | 0.0578 | [H\|ABDF] | 0.05104 |
| 29 | H | 0.4268 | [H\|D] | 0.8999 |
|  | D | 0.3959 |  |  |
|  | DH | 0.156 |  |  |
| 28 | H | 0.3671 | [D\|ABDFH] | 0.1054 |
|  | D | 0.3204 | [H\|ABDFH] | 0.1054 |
|  | DH | 0.2386 | [D\|ABDH] | 0.05976 |
|  |  |  | [H\|ABDH] | 0.05976 |
|  |  |  | [D\|BDFH] | 0.05837 |
|  |  |  | [H\|BDFH] | 0.05837 |
|  |  |  | [DH\|H] | 0.05026 |
| 27 | D | 0.7601 | [D\|BD] | 0.1295 |
|  | H | 0.0944 | [D\|ABDFH] | 0.108 |
|  | DH | 0.0652 | [D\|D] | 0.1041 |
|  |  |  | [D\|ABDH] | 0.09841 |
|  |  |  | [D\|BDFH] | 0.0931 |
|  |  |  | [D\|BDH] | 0.09177 |
|  |  |  | [D\|DH] | 0.06474 |
| 26 | B | 0.4536 | [B\|B] | 0.1682 |
|  | H | 0.2414 | [D\|B] | 0.1044 |
|  | D | 0.1706 | [BD\|B] | 0.08419 |
|  |  |  | [BDH\|B] | 0.07178 |
|  |  |  | [ABDH\|B] | 0.06844 |
|  |  |  | [BDFH\|B] | 0.06427 |
|  |  |  | [DH\|B] | 0.05965 |
|  |  |  | [ABDFH\|B] | 0.0487 |
| 25 | H | 0.5613 | [H\|BDH] | 0.09033 |
|  | B | 0.2296 | [H\|BH] | 0.05847 |
|  | D | 0.0589 | [H\|BD] | 0.05214 |
| 24 | C | 0.9957 | [C\|C] | 0.9984 |
| 23 | C | 0.9752 | [C\|C] | 0.9946 |
| 22 | B | 0.3987 | [BDH\|C] | 0.1935 |
|  | C | 0.3804 | [BH\|C] | 0.1141 |
|  | H | 0.1094 | [ABH\|C] | 0.08688 |
|  |  |  | [BFH\|C] | 0.05522 |
|  |  |  | [ABD\|C] | 0.05203 |
| 1 | B | 0.8387 | [B\|BCH] | 0.1118 |
|  | C | 0.0804 | [B\|ABCH] | 0.05814 |
|  |  |  | [B\|CDH] | 0.05485 |
|  |  |  | [B\|BC] | 0.05386 |
|  |  |  | [B\|BCD] | 0.04974 |
